# Supplementary material for: Development of a minigenome cassette for Lettuce necrotic yellows virus: A first step in rescuing a plant cytorhabdovirus
Source: PLoS One. 2020 Mar 5;15(3):e0229877. doi: 10.1371/journal.pone.0229877 (PMC7058326; doi:10.1371/journal.pone.0229877)
Supplement: S1 Table — Primers were used for PCR amplification for in vitro RNA transcription, cloning, and RT-PCR screening. (DOCX) [file pone.0229877.s001.docx]

| **Primer Set** | **Sense** | **5’ to 3’ sequence** | **Function** |
| --- | --- | --- | --- |
| priV | F | aagacccttcctctatataaggaagttca | Forward primer used to amplify DNA used for *in vitro* transcription |
|  | R | cccttatctgggaactactcacac | Reverse primer used to amplify DNA used for *in vitro* transcription |
| prRN | F | actacatccgctgagaagttg | Sequence specific forward primer used for RT-PCR screening of N gene mRNA. |
|  | R | tgatccaatatcttacctgcc | Sequence specific reverse primer used for RT-PCR screening of N gene mRNA. |
| prRP | F | gatagcgaaagtctcgacttct | Sequence specific forward primer used for RT-PCR screening of P gene mRNA. |
|  | R | actcctccaatgccatcaatg | Sequence specific reverse primer used for RT-PCR screening of P gene mRNA. |
| prRR | F | cttggtatgaggactatcg | Sequence specific forward primer used for RT-PCR screening of DsRed gene mRNA. |
|  | R | tcggttctttcatactgctc | Sequence specific reverse primer used for RT-PCR screening of DsRed gene mRNA. |
| prRint-L | F | gacctatggaatgaagaaacaacg | Sequence specific forward primer used for RT-PCR screening of a sub-segment of int-L gene mRNA. |
|  | R | cagcctttttgtctcagtcct | Sequence specific reverse primer used for RT-PCR screening of a sub-segment of int-L gene mRNA. |
| prin1 | F | aagaacgcatatgactctctgccagactaccatctcca | Exon-sequence specific forward primer used for RT-PCR screening of a sub-segment of int-L gene mRNA containing intron I. |
|  | R | caagtcatcagtttcgtccagtgacaatcggttctcaca | Exon-sequence specific reverse primer used for RT-PCR screening of a sub-segment of int-L gene mRNA containing intron I. |
| prin2 | F | agatccggataggtttctaaggaacacattaaatgatataattgatga | Exon-sequence specific forward primer used for RT-PCR screening of a sub-segment of int-L gene mRNA containing intron II. |
|  | R | catttggatacaccccatgcttctccctgtatccct | Exon-sequence specific reverse primer used for RT-PCR screening of a sub-segment of int-L gene mRNA containing intron II. |
| prin3 | F | agaatgcactcaacaccatagccgggaatgc | Exon-sequence specific forward primer used for RT-PCR screening of a sub-segment of int-L gene mRNA containing intron III. |
|  | R | atccctctgatccgaacctccggttggaac | Exon-sequence specific reverse primer used for RT-PCR screening of a sub-segment of int-L gene mRNA containing intron III. |

**Supplementary Table S1.** **List and description of primers used**. Primers were used for PCR amplification for in vitro RNA transcription, cloning, and RT-PCR screening.
