# Supplementary material for: Development of a minigenome cassette for Lettuce necrotic yellows virus: A first step in rescuing a plant cytorhabdovirus
Source: PLoS One. 2020 Mar 5;15(3):e0229877. doi: 10.1371/journal.pone.0229877 (PMC7058326; doi:10.1371/journal.pone.0229877)
Supplement: S1 Raw images — (PDF) [file pone.0229877.s002.pdf]

Uncropped figures produced in the study are shown here. Red crosses indicate wells not shown in the manuscript

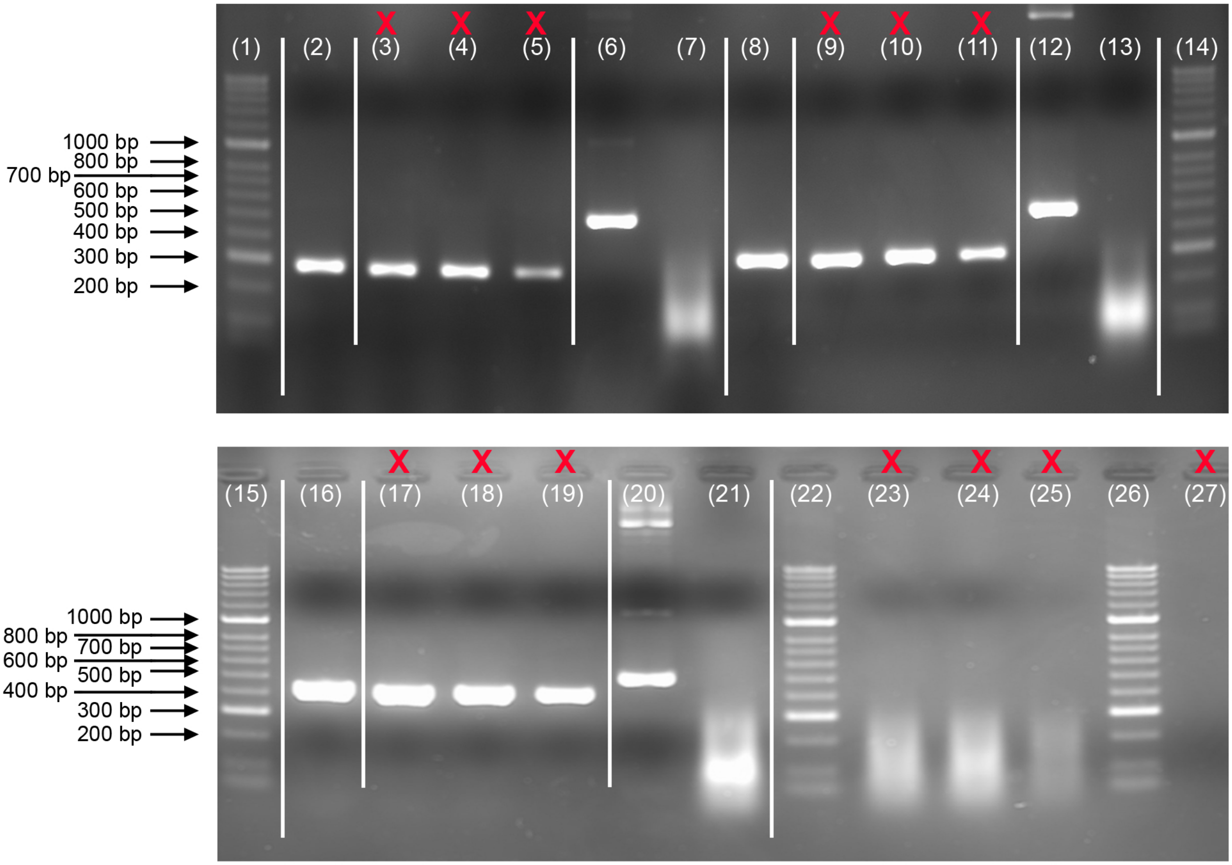

S1_raw_Fig 1: Genome organization of LNYV L gene with or without introduced introns. Agarose gel showing mRNA amplicons detected by RT-PCR compared to PCR of the int-L gene. Lane 1: Hyperladder™ 50 bp DNA Ladder, Bioline. Lane 2: RT-PCR of int-L gene mRNA extracted from *N. glutinosa* using prA set of primers- sample 1 (expected size is 279 bp). Lane 3: RT-PCR of int-L gene mRNA extracted from *N. glutinosa* using prA set of primers - sample 2 (expected size is 279 bp). Lane 4: PCR of L gene sub-fragment (from plasmid) amplified using prA set of primers (expected size is 279 bp). Lane 5: Positive control, RT-PCR amplified L gene using prA set of primers from LNYV inoculated *N. glutinosa* (acquired from DMSZ, Germany) (expected size is 279 bp). Lane 6: PCR of int-L gene (from plasmid) amplified using prA set of primers (expected size is 500 bp). Lane 7: Negative control, RT-PCR of int-L gene mRNA extracted from *N. glutinosa* using prA set of primers without reverse transcriptase. Lane 8: RT-PCR of int-L gene mRNA extracted from *N. glutinosa* using prB set of primers- sample 1 (expected size is 300 bp). Lane 9: RT-PCR of int-L gene mRNA extracted from *N. glutinosa* using prB set of primers - sample 2 (expected size is 300 bp). Lane 10: PCR of L gene sub-fragment (from plasmid) amplified using prB set of primers (expected size is 300 bp). Lane 11: Positive control, RT-PCR amplified L gene using prA set of primers from LNYV inoculated *N. glutinosa* (acquired from DMSZ, Germany) (expected size is 300 bp). Lane 12: PCR of int-L gene (from plasmid) amplified using prB set of primers (expected size is 500 bp). Lane 13: Negative control, RT-PCR of int-L gene mRNA extracted from *N. glutinosa* using prB set of primers without reverse transcriptase. Lane 14: Hyperladder™ 50 bp DNA Ladder, Bioline. Lane 15: Hyperladder™ 50 bp DNA Ladder, Bioline. Lane 16: RT-PCR of int-L gene mRNA extracted from *N. glutinosa* using prC set of primers- sample 1 (expected size is 407 bp). Lane 17: RT-PCR of int-L gene mRNA extracted from *N. glutinosa* using prC set of primers - sample 2 (expected size is 407 bp). Lane 18: PCR of L gene sub-fragment (from plasmid) amplified using prC set of primers (expected size is 407 bp). Lane 19: Positive control, RT-PCR amplified L gene using prC set of primers from LNYV inoculated *N. glutinosa* (acquired from DMSZ, Germany) (expected size is 407 bp). Lane 20: PCR of int-L gene (from plasmid) amplified using prC set of primers (expected size is 500 bp). Lane 21: Negative control, RT-PCR of int-L gene mRNA extracted from *N. glutinosa* using prC set of primers without reverse transcriptase. Lane 22: Hyperladder™ 50 bp DNA Ladder, Bioline. Lane 23: Negative control, total WT *N. glutinosa* RNA RT-PCR amplified using prA set of primers without reverse transcriptase. Lane 24: Negative control, total WT *N. glutinosa* RNA RT-PCR amplified using prB set of primers without reverse transcriptase. Lane 25: Negative control, total WT *N. glutinosa* RNA RT-PCR amplified using prC set of primers without reverse transcriptase. Lane 26: Hyperladder™ 50 bp DNA Ladder, Bioline. Lane 27: Empty lane. Agarose gel was visualised using SynGene G:Box gel imaging technology with Genesys software. Lanes (2), (6) & (7) were combined into int 1 gel of Fig 1 (B). Lanes (8), (12) & (13) were combined into int 2 gel of Fig 1 (B). Lanes (16), (20) & (21) were combined into int 3 gel of Fig 1 (B).

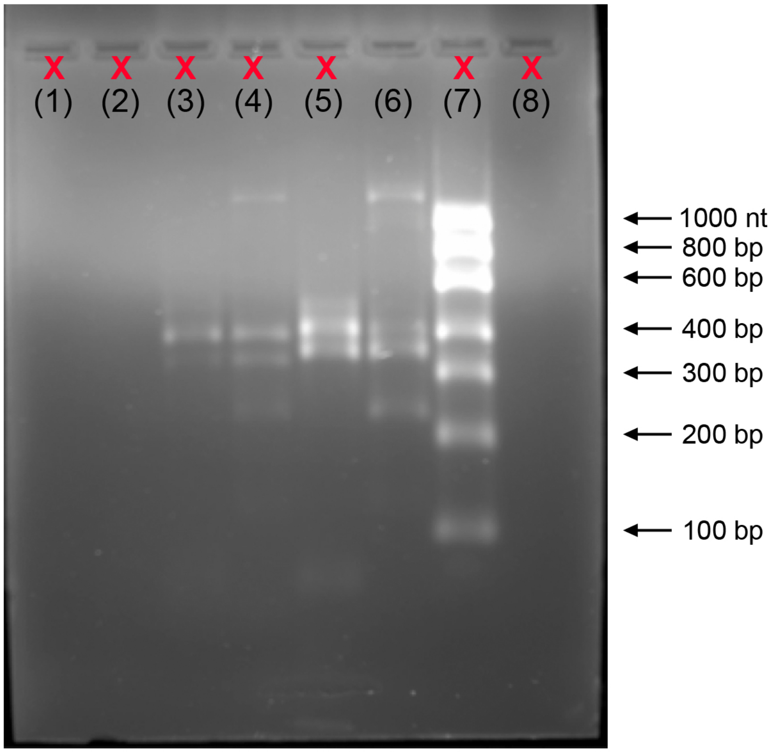

S1_raw_Fig 2 (C): Testing ribozyme auto-cleavage in the LNYV minigenome cassette*.* Lane 1: Empty lane. Lane 2: Empty lane. Lane 3: antigenomic sense minigenome cassette without T7 terminator. Lane 4: antigenomic sense minigenome cassette with T7 terminator. Lane 5: genomic sense minigenome cassette without T7 terminator. Lane 6: genomic sense minigeome cassette with T7 terminator. Lane 7: RiboRuler™ Low Range RNA Ladder, Thermo Scientific. Lane 8: Empty lane. Agarose gel was visualised using SynGene G:Box gel imaging technology with Genesys software. Only lane (6) was used for Fig 2 (C).

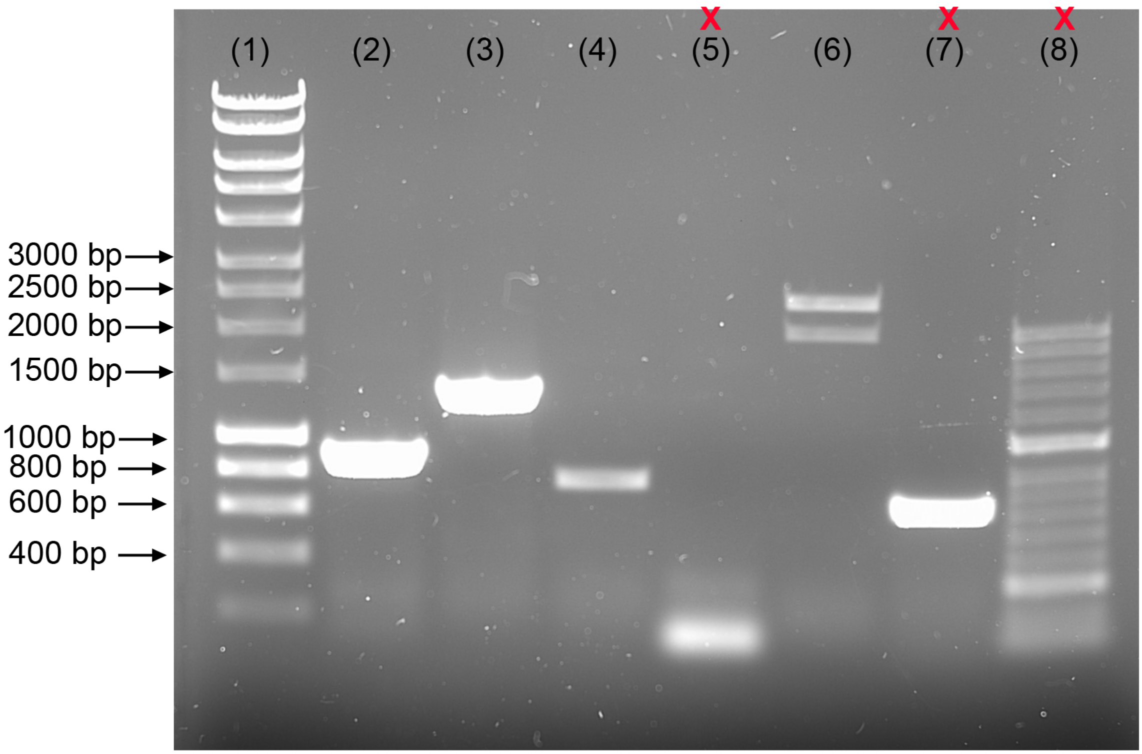

S1_raw_Fig 3 (B): RT-PCR for co-infiltrated genes mRNA. Lane 1: Hyperladder™ 1 kbp DNA Ladder, Bioline. Lane 2: N gene mRNA (expected size 889 bp). Lane 3: P gene mRNA (expected size 1,319 bp). Lane 4: DsRed gene mRNA (expected size is 761 bp). Lane 5: RT-PCR of full Int-L gene mRNA (expected size, excluding introns, is 6207bp). Lane 6: int-L gene mRNA (expected size with all introns spliced is 1,955 bp. expected size with introns I and II is 2,365 bp). Lane 7: Positive control, elongation factor 1 alpha gene mRNA (expected size is 661 bp). Lane 8: Hyperladder™ 50 bp DNA Ladder, Bioline. Agarose gel was visualised using SynGene G:Box gel imaging technology with Genesys software.

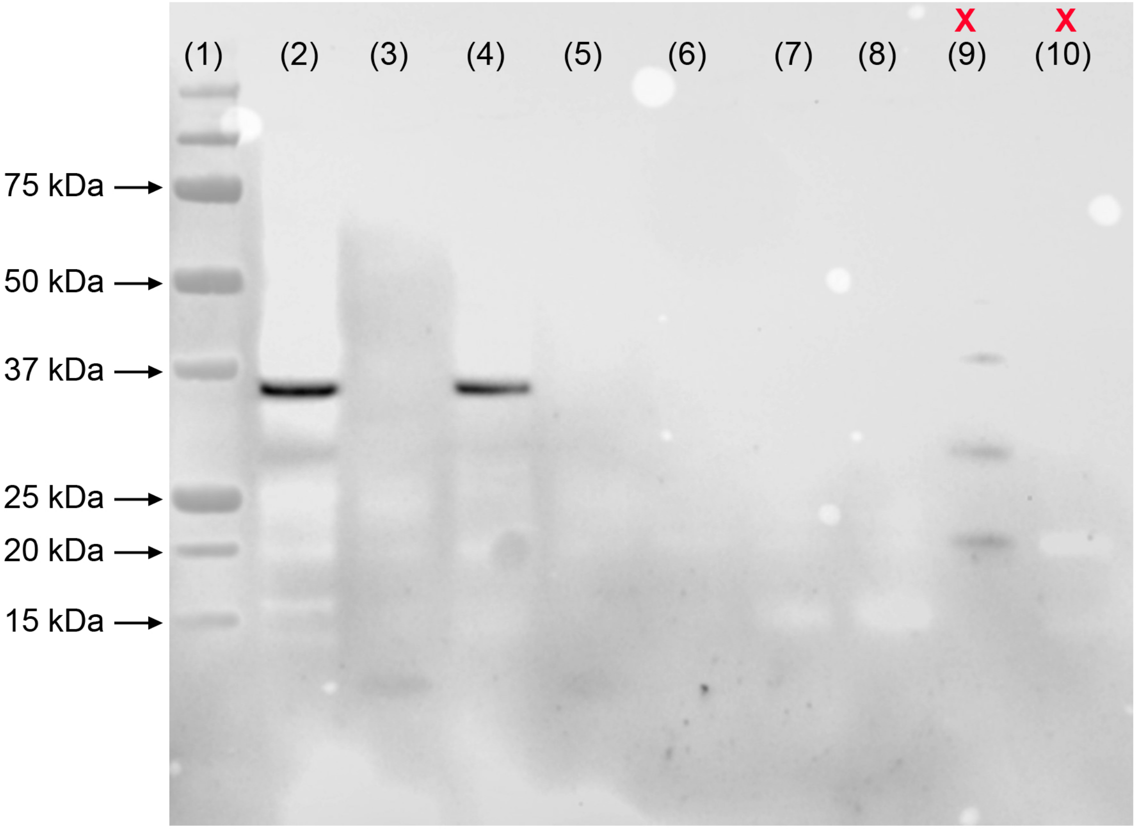

S1_raw_Fig 4 (A): Detection of DsRed in agroinfiltrated *N. glutinosa.* Lane 1: Precision Plus Protein™ Standards, BioRad. Lane 2: MG cassette plus helper proteins (MG+NPint-L). Lane 3: MG cassette alone (MG). Lane 4: Positive control, DsRed with plastid targeting sequence expressed in *N. glutinosa* (R). Lane 5: Negative control (WT non-infiltrated *N. glutinosa*). Lane 6: Helper protein N. Lane 7: Helper protein P. Lane 8: Helper protein int-L. Lane 9: Recombinant DsRed expressed in *E. coli*, Rockland Immunochemicals. Lane 10: Irrelevant *N. glutinoa* extract to run in the lane. Size of DsRed protein with plastid targeting sequence is 33.5 kDa. Size of recombinant DsRed expressed in *E. coli* is 28 kDa. Western blot was visualised using SynGene G:Box gel imaging technology with Genesys software.

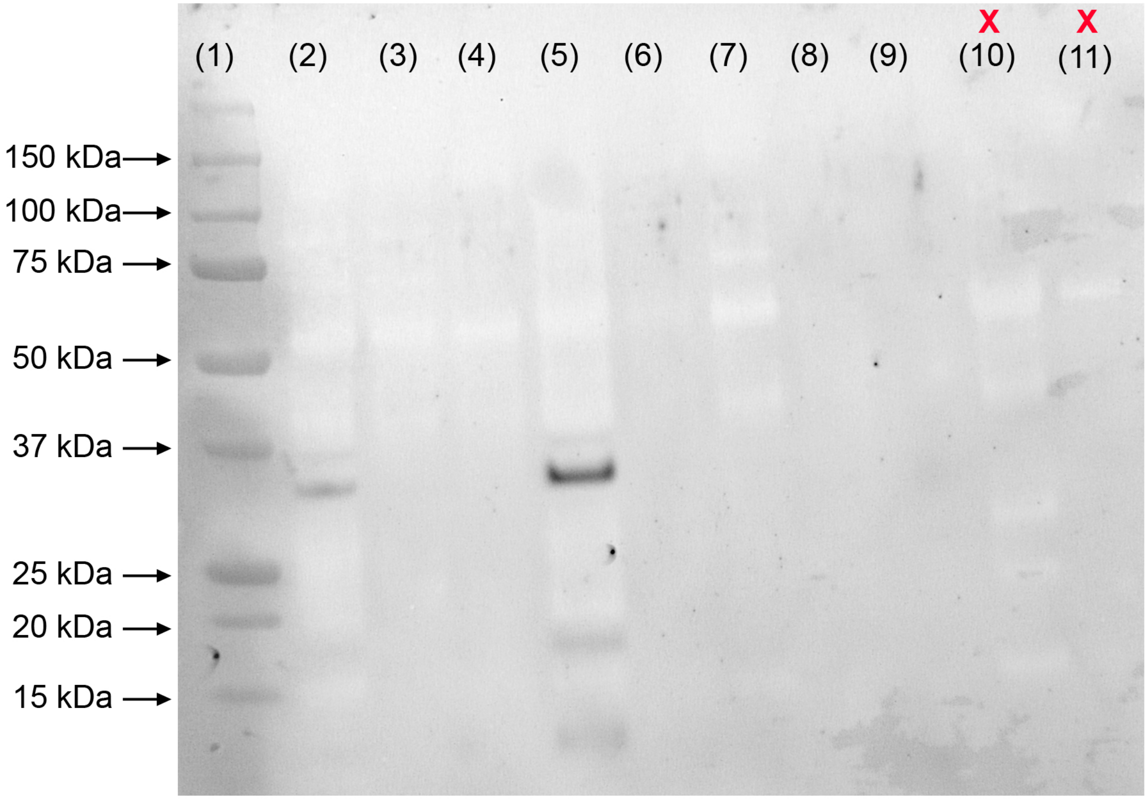

S1_raw_Fig 4 (B): Detection of DsRed in agroinfiltrated *N. benthamiana.*
Lane 1: Precision Plus Protein™ Standards, BioRad. Lane 2: MG cassette plus helper proteins (MG+NPint-L). Lane 3: MG cassette alone (MG). Lane 4: Helper proteins alone (NPint-L). Lane 5: Positive control, DsRed with plastid targeting sequence expressed in *N. benthamiana* (R). Lane 6: Negative control (WT non-infiltrated *N. benthamiana*). Lane 7: Helper protein N. Lane 8: Helper protein P. Lane 9: Helper protein int-L. Lane 10: Irrelevant *N. benthamiana* extract to run in the lane. Lane 11: Irrelevant *N. benthamiana* extract to run in the lane. Size of DsRed protein with plastid targeting sequence is 33.5 kDa. Western blot was visualised using SynGene G:Box gel imaging technology with Genesys software.
