# Supplementary material for: Development of a minigenome cassette for Lettuce necrotic yellows virus: A first step in rescuing a plant cytorhabdovirus
Source: PLoS One. 2020 Mar 5;15(3):e0229877. doi: 10.1371/journal.pone.0229877 (PMC7058326; doi:10.1371/journal.pone.0229877)
Supplement: Attachment — Submitted filename: PONE-D-19-25109_reviewer edit.pdf [file pone.0229877.s003.pdf]

Manuscript

Click here to download Manuscript Ibrahim et al 2019-Manuscript.docx

Development of a minigenome cassette for *Lettuce Necrotic Yellows Virus*: a first

I think it is now accepted that only the species name is italicised - not the actual pathogen?

step in rescuing a plant cytorhabdovirus

Ahmad E. C. Ibrahim[1], Craig J. van Dolleweerd[1], Pascal M.W. Drake[1+], and Julian K-C. Ma[1+]

[1]St. George's University of London, Institute for Infection and Immunity, London, SW17 0RE, United Kingdom

[*] pdrake@sgul.ac.uk

[+] these authors contributed equally to this work

Introduction

Lettuce Necrotic Yellows Virus (LNYV) is a plant cytorhabdovirus with a negative-sense, single-stranded, RNA genome that infects lettuce and garlic (1, 2). *In planta*, the virus replicates within the cytoplasm accumulating in membrane-bounded viroplasms (3, 4). LNYV genomic RNA (gRNA) encodes six monocistronic genes: Nucleoprotein (N), Phosphoprotein (P), cell-to-cell movement protein (4b), Matrix protein (M), Glycoprotein (G), and Large Polymerase (L), flanked by the untranslated 3' *leader* and 5' *trailer* regions (5). As with other members of the *Rhabdoviridae* family, gRNA coiled with N protein acts as the template for *de novo* mRNA synthesis by the viral RNA-dependent RNA polymerase (6). The viral transcription and replication processes are controlled by *cis*-acting sequences in the *leader* (*le*) and *trailer* (*tr*) regions (7-9).

The inherent characteristics of rhabdoviruses, such as having well-defined transcription start/stop signals (10) and the ability to incorporate recombinant glycoprotein into their envelope (11), make them attractive candidates for various biomedical applications. For example, established reverse genetic systems for animal rhabdoviruses (12, 13) enabled rescue of vesicular stomatitis virus (VSV) as recombinant viral vaccine (14, 15). In such systems, cells are transfected with plasmid encoding the viral genome together with the N, P, and L proteins (helper proteins) (16). The viral genome is provided in the positive (antigenomic) sense (17) and auto-cleaving ribozymes such as hammerhead (18) and hepatitis delta (19) are used to increase yield by generating the exact termini of the *de novo* transcribed viral RNA. Recently, this system was adapted for the plant nucleorhabdovirus, *Sonchus Yellow Net Rhabdovirus* (SYNV), where *Agrobacterium tumefaciens* was used to deliver plasmids encoding the antigenomic-sense of the viral genome together with the N, P, and L proteins (20).

only Sonchus should have capital, no italics

Here we attempted to adopt such a system to rescue LNYV. We employed a minigenome (MG) system (21, 22), in which the full-length viral cDNA was substituted by that of a reporter gene

cloned between the viral (*le*) and (*tr*) sequences. Minigenome-based systems are a rapid and simple approach for proof-of-concept studies. As with full-virus RNA transcripts, the MG-derived reporter gene RNA transcripts undergo transcription and replication by the helper proteins. Detection of the reporter protein demonstrates the productive interaction between the viral *cis*- and *trans*- acting elements. We assembled LNYV negative-sense MG cassette with *Discosoma* sp. red fluorescent protein gene (DsRed) cloned in the negative-sense between LNYV *(le)* and *(tr)* sequences and hammerhead (HHz) and hepatitis delta virus (HDz) ribozymes at the 5' and 3' termini respectively, in the plant expression vector pTRAk.2 (23). Similarly, we cloned the N and P genes open reading frame(s) (ORF) into pTRAk.2.

why are these in brackets?

However, our initial attempts to clone the L gene ORF into pTRAk.2 were hampered due to its instability in *E. coli*. Viral sequence instability in *E. coli* has been observed previously where bacteria harbouring plasmids with viral sequences fail to grow or yield plasmids with nucleotide mutations. The reason for this instability remains unknown but different strategies such as cell-free cloning (24) or intron~~s~~ introduction (25, 26) have been employed to circumvent this problem. In the present study, we overcame L gene instability by introducing three introns and using an *E. coli* strain that enable d downregulation of plasmid copy-number.

Following successful molecular cloning of all four constructs, we used *A. tumefaciens* to co-deliver the plasmids into *Nicotiana glutinosa* and *Nicotiana benthamiana*. The former is known to be susceptible to LNYV infection and has been the plant of choice for studying virus biology and pathogenesis while the latter is the species of choice for production of recombinant proteins in plants using *Agrobacterium*-mediated transient expression (27, 28).

Materials and methods

Bacterial strains

Initially, we assembled LNYV *in vitro* MG cassettes to assess ribozyme auto-cleavage in an *in*

surely just tr and le would suffice?

vitro transcription assay. The MG cassette consisted of LNYV *(tr)* – multiple cloning site (MCS) – *(le)* cloned between hammerhead and hepatitis delta and under the control of T7 promoter (Fig. 2A).

As shown in (Fig. 2B), gel electrophoresis of the transcribed products yielded a fragment of expected size for the *(tr)* – MCS – *(le)* sequence (358nt) indicating auto-cleavage by both HHz and HDz ribozymes. Additional bands comprising HHz – *trailer* – MCS – *leader* (412nt) and HDz – T7 (233nt) terminator were also seen.

Fig 2. Testing ribozyme auto-cleavage in the LNYV minigenome cassette. (**A**) Diagram of construct used in T7 *in vitro* RNA transcription showing the LNYV minigenome cassette (*tr)* – MSC - (*le)* flanked by hammerhead and hepatitis delta ribozymes for generating exact viral termini by auto-cleavage. The minigenome cassette is flanked by the T7 promoter and terminator for *in vitro* transcription. (**B**) Agarose gel showing the expected 385 nt (*tr)* – MCS - (*le)* fragment plus 412 nt HHz - *(tr)* – MCS - *(le)* and 233 nt HDz - T7T bands.

We next sought to test the LNYV MG cassette *in planta* to assess the interaction between LNYV *cis*- and *trans*- acting elements. We substituted the MG MCS with the DsRed gene ORF, cloned in the negative-sense with a plastid targeting sequence at the N – terminus. The plastid targeting sequence was employed to minimize metabolic burden on the endoplasmic reticulum

Was cytoplasmic localisation tested? Why not, if not?

(36). The coding region was cloned between the 3' and 5' UTRs from LNYV N and L genes respectively. The construct was cloned into the pTRAk.2 plant expression plasmid (Fig. 3A) under the control of the cauliflower mosaic virus 35S double promoter for transient expression in *Nicotiana* (37). N, P and int-L genes were cloned in separate plasmids.

N. glutinosa leaves were co-infiltrated with the MG cassette, N, P and int-L. 7 days post-infiltration, total RNA was extracted from co-infiltrated leaf samples and used as a template for RT-PCR using N, P, int-L, and DsRed gene-specific primers (sets prRN, prRP, prRint-L and prRR

Fig 4. Detection of DsRed in agroinfiltrated *Nicotiana*. (**A**) DsRed protein detection using western blot in agro-infiltrated *N. glutinosa*. Infiltration was with MG cassette plus helper proteins (MG+NPint-L), MG alone (MG), helper proteins alone (N), (P), (int-L) and the positive control (R). WT is wild type non-infiltrated *N. glutinosa*. Bands were detected in samples infiltrated with MG cassette plus helper proteins (MG+NPint-L) and in positive control (R). (**B**) DsRed protein detection using western blot in agro-infiltrated *N. benthamiana*, samples are as in (**A**) but with an additional negative control (NPintL) consisting of infiltration of all helper proteins.

Similarly, western blotting of crude *N. benthamiana* extract samples confirmed the presence of DsRed protein in samples infiltrated with MG cassette, N, P and int-L plasmids (Fig. 4B, lane mrR+NPint-L) and in the positive control (lane R). No bands were seen in *N. benthamiana* infiltrated with MG cassette only (lane MG), or in samples infiltrated with individual helper plasmids (lanes MG, N, P, and int-L respectively) or in non-infiltrated sample (lane WT). A further negative control consisting of sample infiltrated with all three helper plasmids but without MG was included here and did not produce DsRed expression (lanes NPint-L).

Discussion

In the present study, we have investigated the feasibility of a reverse genetic system for LNYV based on a negative-sense MG cassette. The MG-derived DsRed RNA-transcripts underwent transcription and expression in plant cells co-expressing N, P, and L proteins. Reverse genetic systems based on positive-sense constructs have been long established for animal and plant rhabdoviruses (12, 13, 22). However, to the best of our knowledge, only two negative-sense RNA viruses have been rescued from negative-sense constructs: rabies and Sendai virus (17, 38). The advantage of reverse genetic systems based on negative-sense constructs is that it excludes any possibility of MG cassette basal expression independent of the N, P, and L proteins.

The MG cassette together with the genes of the three helper proteins: N, P, and L were independently cloned into pTRAk.2 vector and were agro-infiltrated into *N. glutinosa* and *N. benthamiana* using *A. tumefaciens*. Cloning the L gene in *E. coli* was initially problematic. Difficulty in cloning RNA virus genomes or viral genes in *E. coli* is common among the RNA viruses (24, 39-41). The presence of prokaryotic promoters in viral sequences and spurious activity of eukaryotic promoters are found to be contributing factors for the observed instability (42, 43). Strategies including the use of single point mutations, insertion of introns, and use of low-copy plasmid have been used to overcome these problems (26, 44, 45). Nevertheless, success is predominantly a matter of trial and error as demonstrated by attempts to clone dengue virus type 1 (DV1) where a low-copy plasmid approach was suitable for cloning a DV1 replicon, but failed to produce the full-genome DV1 cDNA (46). Ultimately, we succeeded in cloning the full-length L gene by incorporating three different introns into the 5' half of the gene and using an *E. coli* strain with modified *pcnB* gene for reduction of plasmid copy number (47). While all three chosen introns have previously been demonstrated to be correctly spliced in their plant of origin (33-35), none have previously been tested as part of a cytorhabdovirus genome or in *N. glutinosa*. Insertion sites for introns were chosen between thymine and guanine nucleotides of ATG as described previously (25). Introns were inserted at the 5' end of the gene to further enhance *in planta* mRNA translation (48). Attempts to clone the full-length L gene using an intron-only approach (i.e., without plasmid copy number reduction), or solely by plasmid copy number reduction (i.e., without incorporation of introns) were unsuccessful. A possible explanation is that disruption of cryptic sequences by introns and low template number (plasmid copy number) minimized inadvertent transcription (49). Post-agroinfiltration, RT-PCR assessment performed on total RNA revealed the correct splicing of all three introns to yield mature mRNA, which was further confirmed by cDNA sequencing.

The first assessment undertaken with the assembled MG cassette was to confirm ribozyme auto-cleavage. The generation of exact 5′ and 3′ termini of vRNA is critical for transcription and
