# Supplementary material for: Development of a minigenome cassette for Lettuce necrotic yellows virus: A first step in rescuing a plant cytorhabdovirus
Source: PLoS One. 2020 Mar 5;15(3):e0229877. doi: 10.1371/journal.pone.0229877 (PMC7058326; doi:10.1371/journal.pone.0229877)
Supplement: Attachment — Submitted filename: Response to Reviewers.docx [file pone.0229877.s004.docx]

We thank the editor and the reviews for their comments and suggestions. Below are our answers to questions raised either in the text of the email sent on 04/12/2019 or the attached PDF.

**5. Review Comments to the Author**

Please use the space provided to explain your answers to the questions above. You may also include additional comments for the author, including concerns about dual publication, research ethics, or publication ethics. (Please upload your review as an attachment if it exceeds 20,000 characters)

Reviewer #1: The paper does not have any major scientific flaws but does not provide any interesting information either. The work presented has been done before alas not with LNYV. Is this good enough to warranty publication in PLoSOne? Not in my opinion. It just lacks originality.

We respectfully disagree with this statement. In terms of manuscript originality, this is the first time a minigenome cassette strategy has been developed for LNYV. The Sonchus yellow net rhabdovirus (SNYV) publication (Wang et al. 2015) was based on a positive-sense construct, whilst our work in the present paper is based on negative-sense construct. In addition, SYNV is a nucleorhabdovirus whereas LNYV is a cytorhabdovirus. Finally, we believe that our strategy for solving viral gene instability in *E. coli* will be of considerable benefit to researchers.

Reviewer #2: The paper by Ibrahim and co-authors describes the set up of a replicating minigenome cassette based on genomic components of a cytorhabdovirus, lettuce necrotic yellows virus.

The paper is well written, with a well thought experimental plan, and the main conclusion seem to be well supported by the experiments carried out.

It needs to be improved a little bit, with further explanations that would make this paper useful for repeating experiments and future planning of the same work by other laboratories.

Thank you for the supportive comments. We address your comments below and in the manuscript.

Here are my suggestions for improving it:

Line 19: maybe better “membrane-bound”? This has been changed as requested.

Line 32: plasmids This has been changed as requested.

Line 59-60: are you sure N. glutinosa is the choice plant species for studying virus biology and pathogenesis? The same can be said for N. benthamiana. Yes, *N. glutinosa* is the plant species of choice for this virus. In addition to the publications cited in our manuscript, others include: (1) B. Harrison and N. Crowley, "Properties and structure of lettuce necrotic yellows virus," *Virology,* vol. 26, no. 2, pp. 297-310, 1965. or (2) T. Chambers and R. Francki, "Localization and recovery of lettuce necrotic yellows virus from xylem tissues of Nicotiana glutinosa," *Virology,* vol. 29, no. 4, pp. 673-676, 1966. or (3) B. Wolanski, R. Francki, and T. Chambers, "Structure of lettuce necrotic yellows virus: I. Electron microscopy of negatively stained preparations," *Virology,* vol. 33, no. 2, pp. 287-296, 1967.

We have included *N.benthamiana* as it is the species of choice for plant molecular farming using transient expression and believe that a positive result in the two species strengthens the paper.

Line 140: N. glutinosa in Italics. This has been changed as requested.

In general, I think you need to specifically show in a figure (figure 2) a panel that shows the HHz-ribozyme sequence, with the initial viral sequence, and the putative cleaving site. This is important to show, and it would be better to show the secondary structure. No need to do so for the HDz). We have added this as requested. We have also added “The MG cassette consisted of tr – multiple cloning site (MCS) – le cloned between hammerhead and hepatitis delta and under the control of T7 promoter (Fig. 2A). We also changed the first 8 nucleotides of HHz (accession number GU299211) to 5’‑UCGUCCGU-3’, to allow base-pairing with the first 8 nucleotides of the LNYV tr (5’‑ACGGACGA-3’) as indicated in a previous study (18)” to the manuscript.

Having said that, what you show in Fig.2.B is some evidence of cleaving (but not of the exact cleaving). Furthermore, cleaving is predicted based on RNA sizes (difficult to estimate in agarose gel, likely non-denaturing, even if not specifically stated in the Fig. 2 legend). So I would extensively use the word “putative” to describe the band assigned to the fragments predicted. We have added ‘putative’ into number of places in the manuscript including in the Fig2 legend. “(**C**) Agarose gel showing putative bands of tr – MCS - le fragment (358 nt), HHz - tr – MCS - le (412 nt), and HDz - T7T (233 nt).”

Line 174-175: it would be better to specifically mention the exact nt start and end of the 5’ and 3’ UTR referred to a GenBank reference viral sequence. This has been changed as requested. “The coding region was cloned between LNYV L gene 3’ UTR (extending between 12,564 and 12,613nt of LNYV genome accession number AJ867584) and N gene 5’ UTRs (extending between 92 and 168nt).”

Line 251: specify better in which sense they were “unsuccessful” This has been specified as requested. “transformed *E. coli* either did not grow or yielded plasmids with large deleted sequences, although a number of strains were used with different growth conditions)”

Line 259-262: as I said above, you should have used northern blots with specific probes to identify better each of the three bands. I think this part of the discussion needs to be tuned down, or erased. We have modified this line as follows: “Gel electrophoresis of MG RNA transcribed *in vitro* at 25°C suggested ribozyme auto-cleavage at both termini with the putative HDz auto-cleavage being more rapid than that of the HHz (which contradicts the results of previous studies (22)). Variation in ribozyme efficacy could have occurred due to differences in incubation temperature (52), and in future studies northern blotting with specific probes should be used better identify the bands.

278-281: I am a little surprised about this. Probably working with appropriate filters and positive controls, you should have been able to distinguish dsRED from Autofluorescence. And even if I am not specifically asking for it, it would have been good to also have a GFP construct. Visual aspect of infection are also important, fur future steps when you will add putative movement proteins.

We agree with this statement. DNA sequencing confirmed that the dsRED construct was correct - low expression levels in the plant may have been the reason for our difficulty in distinguishing between dsRed fluorescence and autofluorescence. Unfortunately, time and funding constraints prevented further exploration, however this will be a priority in future studies. We do believe that the western blotting results presented are conclusive.

The arrows in Fig 1 are too big and misleading in some way. They need to be on the sides of the intron sites…. Not across the intron side. This has been changed as requested.

Fig 4 should show some loading control (Coomassie or poinceu red).

Unfortunately, we are do not have these data, however assessments were undertaken with leaf samples of the same mass treated under identical conditions.

Reviewer #3: Nice ms, describes difficult work that was well done, and of significant interest both to plant virologists and to plant molecular biotechnologists. I have very few concerns, and those are mainly use of taxonomic terms (see annotated ms). The English was excellent, the work was well described, and the results good enough to merit publication.

Thank you for these supportive comments.

**From the reviewer notes in attached PDF**

Page 9:

Surely just tr and le would suffice?

A minimal construct, based on ribozymes together with the leader and trailer sequences will suffice for the *in vitro* investigation. However, the MCS was necessary as it made it possible to clone the reporter gene for later *in vivo* experiments.

Was cytoplasmic localisation tested? Why not, if not?

No, cytoplasmic localisation was not tested. Unfortunately, due to restricted time and funding no cytoplasmic localisation experiments could be conducted but this will be a priority in future studies.
